# The Threshold Photoelectron Spectrum of the Vinylcyclopentadienyl Radical, a C_7_H_7_ Resonance-Stabilized Radical

**DOI:** 10.1021/acs.jpca.6c02174

**Published:** 2026-06-09

**Authors:** Rory McClish, Megan R. Bentley, Mia Muse, Gregory H. Jones, Peter R. Franke, Domenik Schleier, Patrick Hemberger, Andras Bodi, G. Barney Ellison, John F. Stanton, Jordy Bouwman

**Affiliations:** † Laboratory for Atmospheric and Space Physics, 1877 University of Colorado, Boulder, Colorado 80303, United States; ‡ Department of Chemistry, University of Colorado, Boulder, Colorado 80309, United States; § Quantum Theory Project, Department of Chemistry, 3463 University of Florida, Gainesville, Florida 32611, United States; ∥ Institut für Physik und Astronomie, 199914 Technische Universität Berlin, Hardenbergstrasse 36, Berlin 10623, Germany; ⊥ Laboratory for Synchrotron Radiation and Femtochemistry, Paul Scherrer Institute, 5232 Villigen, Switzerland

## Abstract

We generated the aromatic resonance-stabilized vinylcyclopentadienyl radical, C_5_H_4_–CH=CH_2_, in the gas phase via flash pyrolysis of *meta*-vinylanisole, CH_3_O–C_6_H_4_–CH=CH_2_. Double-imaging photoelectron photoion coincidence spectroscopy was used to measure the photoion mass-selected threshold photoelectron spectrum of the vinylcyclopentadienyl radical. Spectral assignments were enabled by independent, high-level *ab initio* coupled cluster calculations of adiabatic ionization energies originating from the ground electronic state of the doublet neutral, X̃ ^2^A″, to both the ground state of the cation, X̃^+ 1^A′, and the lowest triplet state, ã^+ 3^A′. The experimentally determined band origin energies are found to be (7.81 ± 0.02) eV (X̃^+ 1^A′ ← X̃ ^2^A″) and (8.08 ± 0.02) eV (ã^+ 3^A′ ← X̃ ^2^A″), in agreement with the respective calculated values of (7.83 ± 0.01) eV and (8.09 ± 0.01) eV. The combined experimental and theoretical characterization presented here provides a foundation to identify important resonance-stabilized radicals in complex gas-phase environments.

## Graphical abstract

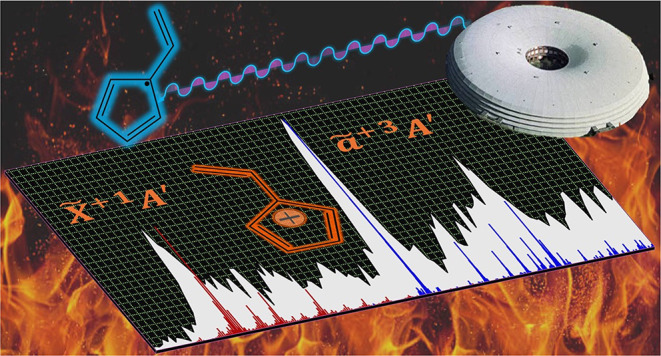

## Introduction

Resonance-stabilized hydrocarbon radicals, RSRs, are ubiquitous across reactive gas-phase environments spanning combustion^1,2^ and interstellar contexts.^3,4^ The degree of resonance stabilization conferred by electron delocalization is referenced to the difference in the R–H bond dissociation energy between the closed-shell RSR precursor molecule and methane.^5^ Due to their energetic stability, RSRs are often found in appreciable abundances; hence RSR reactions are important for consideration in molecular growth schemes including the formation of (poly)­cyclic aromatic hydrocarbons.^1,6^

Radical-driven chemistry plays an important role in soot formation.^1^ The initial stage, soot inception, includes the production of the gas-phase precursors that then transition to the condensed phase.^7^ Johansson et al.^8^ analyzed the molecular composition of soot particles from flames using vacuum ultraviolet aerosol mass spectrometry (VUV-AMS). Odd-valued peaks in their mass spectra up to about 263 u implicated successive radical chain chemistry, leading them to propose that clustering of hydrocarbons by radical-chain reactions (CHRCR) explains soot formation.^8^ The photoionization onset of *m*/*z* 91 was inconsistent with the most thermodynamically stable C_7_H_7_ isomer benzyl (C_6_H_5_–CH_2_·), as well as the cycloheptatrienyl radical (tropyl, *c*-C_7_H_7_·), both of which are RSRs. The experimental onset near 7.9 eV agreed with calculated adiabatic ionization energies (AIE) of 7.94 (ROCBS-QB3) and 7.81 eV (ωB97X-D/aug-cc-pVTZ) of the vinylcyclopentadienyl radical (C_7_H_7_, C_5_H_4_–CH=CH_2_). In a later study, Jin et al.^9^ monitored the total ion signal as a function of photon energy to measure the *m*/*z* 91 photoionization (PI) spectrum resulting from the pyrolysis of *para*-vinylanisole (i.e., 4-methoxystyrene, CH_3_O–C_6_H_4_–CH=CH_2_). They reported the photoionization onset of the 91 u product to be 7.84 eV and assigned the isomer as vinylcyclopentadienyl based on the same calculated AIEs referenced above. This vinylcyclopentadienyl PI spectrum was used in subsequent VUV-AMS work by Rundel et al.^10^ to disentangle the C_7_H_7_ isomers in soot particles produced from a variety of parent fuels under a range of physical conditions. More recently, the comparison study by Shahanand et al.^11^ of the gas-phase pyrolysis of both bare anisole and *para*-vinylanisole decomposition included a measurement of the PI spectrum of vinylcyclopentadienyl consistent with Jin et al.^9^ and reported the ionization energy at (7.82 ± 0.02) eV. Semiautomated electronic structure calculations of the C_7_H_7_ potential energy surface (cognizant of the combinatorial issue of ∼1000 possible isomers) found that the three lowest-energy C_7_H_7_ isomers—benzyl, tropyl, and vinylcyclopentadienyl—are separated by relatively high energy barriers and are thus largely kinetically isolated from one another.^12,13^ Taken with the VUV-AMS results of Rundel et al.,^10^ this suggests that it is important to consider isomer-specific chemistry of C_7_H_7_ RSRs in developing predictive models of soot inception chemistry.

In complex mixtures it becomes difficult to resolve isomeric contributions in PI spectra.^14^ Photoion mass-selected threshold photoelectron (ms-TPE) spectroscopy based on double imaging photoelectron photoion coincidence (*i* ^2^PEPICO) measurements allows for resolving vibronic transitions as peaks, enabling definitive spectroscopic assignments.^15–20^ Increasingly, *i* ^2^PEPICO is being used as a quantitative analytical tool for studying molecular growth and soot inception.^21–23^ In the case of C_7_H_7_, the ms-TPES of both benzyl^24^ and tropyl^25^ radicals have been measured. However, the ms-TPE spectrum (ms-TPES) of the vinylcyclopentadienyl radical has not been reported yet. Moreover, both benzyl and tropyl are known to exhibit large singlet–triplet (S–T) energy splittings (ΔS–T ≈ 2.2, 3.4 eV, respectively). In contrast, the (unrestricted) CBS-QB3 calculations of Savee et al.^26^ predict a much smaller S–T splitting of 0.19 eV for vinylcyclopentadienyl. Considering that the typical uncertainty of CBS methods is on the order of 1 kcal/mol (≈43 meV),^27^ it is expected that this may limit the ability of such methods to provide definitive assignments. A resolved PI spectrum may indicate the triplet state in the form of a step-like feature, but only a subtle change in the slope is seen in the previously measured PI spectra,^9,11^ leaving experimental identification of the triplet state uncertain so far.

In this work, we report the ms-TPES of the vinylcyclopentadienyl radical and assign the band origins based on extensive quantum chemical calculations. Similar to Jin et al.^9^ and Shahanand et al.,^11^ we pyrolyze vinyl-substituted anisole to generate vinylcyclopentadienyl. We apply fully *ab initio* computations rooted in coupled cluster theory, with particular care directed toward the inclusion of high orders of electron correlation, in the pursuit of sub-kJ/mol accuracy of computed adiabatic ionization energies and singlet–triplet splittings. By combining isomer-selective ms-TPES measurements with state-of-the-art electronic structure calculations, this work provides the first definitive photoelectron spectroscopic characterization of the vinylcyclopentadienyl radical.

First we describe the experimental and computational methods. Next, we report the ms-TPES of the 91 u species formed upon pyrolyzing *meta*-vinylanisole. Subsequently, we present the quantum chemistry calculation results and assign the spectrum. Finally we discuss the S–T gap of the vinylcyclopentadienyl cation in relation to the cyclopentadienyl radical, as well as within the context of isomer-resolved detections of C_7_H_7_ mixtures.

## Methods

### Experimental Methods

Pyrolysis experiments were conducted using a heated microreactor coupled to the double imaging photoelectron photoion coincidence (*i* ^2^PEPICO) spectrometer endstation at the vacuum ultraviolet (VUV) beamline of the Swiss Light Source (SLS) at Paul Scherrer Institute, Villigen. The *i* ^2^PEPICO experiment and beamline details have been described previously^28–30^ and only a brief discussion of relevant parameters is provided here.

*Meta-*vinylanisole (**mVA**, CH_3_O–C_6_H_4_–CH=CH_2_) was purchased from Sigma–Aldrich (≥97%) and kept at room temperature in a glass bubbler. A flow of argon gas was regulated by an MKS mass flow controller to 10–14 sccm before flowing through the bubbler to pick up **mVA** vapor (*P*_vap__*p*_ = 1.3 mbar at 316 K).^31^ The resulting gas mixture entered the source vacuum chamber via a stainless steel feedline tube, in which the pressure was measured to be between 220 and 300 mbar. The concentration of **mVA** in Ar is thus estimated at ∼0.5%. At the end of the feedline tube, the gas expanded out of a 100 μm diameter pinhole into a ∼3 cm long SiC carbide microtubular reactor with an inner diameter of 1 mm mounted inside the source vacuum chamber. A DC current was passed through two electrodes fixed approximately 2 cm apart to resistively heat the microreactor. Reported wall temperatures are based on a previous calibration as a function of the dissipated power with an uncertainty of ca. ± 100 K. Further details of such Chen-type SiC microreactors have been explored previously.^32–34^

Upon exiting the SiC microreactor, the gas expanded into the source chamber maintained at ∼1–5 × 10^–4^ mbar by two 1600 Ls^–1^ Pfeiffer turbomolecular pumps. The expansion was skimmed by a 1 mm diameter skimmer to form a molecular beam in the adjacent detection chamber, which was maintained at a pressure of 5–10 × 10^–7^ mbar. In the detection chamber, the molecular beam was intercepted by tunable VUV synchrotron radiation generated using a bending magnet and subsequently monochromatized using a 150 grooves/mm gold-coated blazed grating. A MgF_2_ window suppressed higher harmonics above 10.5 eV. The resulting photon energy resolution at the endstation was 1:1500, i.e., on the order of 5 meV at a photon energy of 8 eV at a total monochromatic flux of ca. 10^11^ photons/s.

Species ionized by the tunable VUV radiation form pairs of photoelectrons and photoions. These were accelerated in opposite directions by an electrostatic field of 230 V/cm and imaged onto two RoentDek delay-line detectors. The photoelectron–photoion pairs were detected in delayed coincidence. Photoionization time-of-flight mass spectra (PI-TOF-MS) were constructed at single photon energies, with typical integration times of 1 min. Mass-selected total ion yield photoionization spectra can be extracted by scanning the photon energy and considering coincidences of photoelectrons, regardless of their kinetic energy, and ions in an *m*/*z* channel. Electrons are imaged onto the detector quantitatively when their energy is up to ca. 700 meV (see Figure S6). Photoion mass-selected *threshold* photoelectron spectra were constructed by further restricting photoelectron energies to near-zero, thus selecting for the internal energy of the photoions. This was achieved by analyzing ion counts coincident with photoelectrons at the center of the electron-detector image. Contributions from “hot” electrons, i.e., electrons with larger (non-threshold) kinetic energies that were ejected perpendicular to the plane of the detector and also landed on the center of the electron image, were corrected for as described by Sztáray and Baer.^35^ Plotting the ion counts of a particular *m*/*z* coincident only with threshold photoelectrons as a function of photon energy produces the mass-selected threshold photoelectron spectrum (ms-TPES). The spectra reported here are not corrected for variations in photon flux as a function of photon energy. As discussed in prior publications,^36,37^ the Stark effect of ca. 10 meV arises from the constant extraction field and can both asymmetrically broaden bands to the red and shift peak maxima to lower energies; however, this is in part counteracted by the fact that we accept electrons with kinetic energies of up to 4 meV as threshold counts. The interplay of the Stark effect and electron kinetic energy dependence is expected to result in a minor (ca. 5 meV) shift for most organics.^36^ Here the spectral features are expected to be virtually unaffected by the extraction field and the resolution of the photon and electron energies compared to the rotational broadening and hot bands present at 300 K. The experimental ms-TPE spectra are thus reported as is and no corrective shifts are applied. Rather, the net 20 meV uncertainty associated with experimentally measured ionization energies is conservatively derived from the peak width as described in the Results section.

In the *i* ^2^PEPICO experiment, imaging both photoelectrons and photoions enables the construction of an ms-TPES for ions with a specific momentum distribution. Ions formed from the molecular beam have high velocity in the direction of the expansion with small velocity components perpendicular to the beam, which is resolved on the ion detector. In contrast, there is an isotropic and broad velocity distribution centered about zero average velocity, which corresponds to the background gas that has been scattered and rethermalized within the detection chamber. The ms-TPES can be extracted individually for the molecular beam and the background gas based on the separable velocity distributions of ions from the molecular beam vs those from the background gas (see Hemberger et al.^38^ for a detailed analysis). Consistent with previous microreactor experiments,^36,38–40^ inefficient (ro)­vibrational cooling was observed in the ions sampled from the molecular beam, broadening the ms-TPES. However, the room-temperature background signal displayed rich vibrational fine structure. The contributions of different regions (beam vs background) of the ion detector to the total-detector ms-TPES of *m*/*z* 91 are shown in Figure S4. Here in the main text *m*/*z* 91 ions originating from the rethermalized background gas were selected.

### Computational Methods

All electronic structure calculations were performed within the CFOUR program package.^41–43^ Equilibrium structures of the X̃ ^2^A″, X̃^+ 1^A′, and ã^+ 3^A′ electronic states of C_7_H_7_ were optimized with frozen-core coupled-cluster singles, doubles, and perturbative triples (CCSD­(T))^44–46^ and an atomic natural orbital (ANO1) basis set^47^ (Cartesian coordinate geometries are given in Table S5 of the SI). Due to significant spin contamination in the neutral radical, restricted open-shell Hartree–Fock (ROHF) reference wave functions were employed for the X̃^2^A″ and ã^+3^A′ states, while the closed-shell X̃^+1^A′ state was treated using an RHF reference. Franck–Condon simulations at 300 K, at 0 K) involving the X̃^+ 1^A′ ← X̃ ^2^A″ and ã^+ 3^A′ ← X̃ ^2^A″ transitions, were performed using the FC^2^ package within CFOUR.^41,48^ All modes are included in the simulations.

#### Determination of AIEs by Composite Methods

Adiabatic ionization energies (AIEs) were computed using a composite electronic structure scheme designed to balance high-order electron correlation effects with computational feasibility, represented by eq 1.

1 $$E=E_{\mathrm{HF}‐\mathrm{SCF}}+\Delta E_{\mathrm{fc}‐\mathrm{CCSD}(T{)}_{\Lambda }}^{\infty }+\Delta E_{\mathrm{fc}‐\mathrm{CCSDT}‐\mathrm{fc}‐\mathrm{CCSD}(T{)}_{\Lambda }}+\Delta E_{\mathrm{fc}‐\mathrm{CCSDT}(Q{)}_{\Lambda }}+\Delta E_{\mathrm{CV}}+\Delta E_{\mathrm{REL}}+\Delta E_{\mathrm{DBOC}}+\Delta E_{\mathrm{ZPVE}}$$

The first five terms in eq 1 comprise the nonrelativistic electronic energy, which is then corrected by relativistic (REL), diagonal Born–Oppenheimer (DBOC), and zero-point vibrational energetic (ZPVE) effects. The first term (*E*_HF‑SCF_) is calculated at the HF-SCF level with the cc-pCV6Z basis set of Dunning and co-workers.^49,50^ The HF-SCF contribution to the ionization energy is not extrapolated, as it has been shown in previous work^51,52^ that the quintuple-zeta HF-SCF contribution alone to a total atomization energy is an improvement over a 3-point extrapolated value that includes smaller basis sets that are quite far from convergence. While the previous works use aug-cc-pCVXZ basis sets and are focused on computing total atomization energies, it is reasonable to expect that the more robust computation of an ionization energy is sufficiently well-converged by cc-pCV6Z. Indeed, the AIE increment between cc-pCV5Z and cc-pCV6Z is only 10 cm^–1^ (1.24 meV) for transitions to the lowest two electronic states of the cation, and is <1 cm^–1^ for the cationic singlet–triplet gap, well below the quoted uncertainty of the final adiabatic ionization energies.

All correlation contributions, unless otherwise noted, are evaluated within the frozen-core (fc) approximation, due to the insurmountable computational expense required to carry out all electron (ae) computations with a sufficiently large basis set (pVQZ and beyond) on a molecule of this size. The bulk of the correlation energy is accounted for by the second term, Δ*E*_fc‑CCSD(T)_Λ__^∞^, (abbreviated as Δ*E*_fc‑(T)_Λ__^∞^). This consists of a CCSD­(T)_Λ_ computation^46,53,54^ extrapolated to the complete basis set (CBS) limit through the Helgaker formula^55^ applied to the cc-pVQZ and cc-pV5Z basis sets (denoted in curly braces as pV­{Q,5}­Z). The third term corrects for any deficiencies in the perturbative evaluation of triple excitations by CCSD­(T)_Λ_ by inclusion of a fully iterative treatment CCSDT^56–58^ using the ANO1 basis set. Higher-level correlation effects are accounted for by inclusion of a CCSDT­(Q)_Λ_ ^52,59–61^ – CCSD­(T)_Λ_ correction (Δ*E*_fc‑CCSDT(Q)_Λ__) using an ANO0 basis set.

To recover any missing core–valence correlation (ae–fc), the fc-CCSD­(T)_Λ_ term is corrected by the Δ*E*_CV_ term. This term is constructed from two components, (i) a two-point extrapolated (cc-pCV­{T,Q}­Z) CCSD contribution and (ii) a *E*_CCSD(T)_Λ__ computation with the cc-pCVTZ set.

The one-electron variant of the spin-free exact two-component (SFX2C1e) technique^41,62^ (and references therein) at the ae-CCSD­(T)/cc-pCVTZ-unc level is used to quantify scalar relativistic effects. The diagonal Born–Oppenheimer correction^63–65^ is evaluated at the fc-CCSD/pVTZ level. Finally, zero-point vibrational effects are evaluated at the harmonic level using fc-CCSD­(T)/ANO1, and an additional VPT2 contribution evaluated at fc-CCSD­(T)/ANO0 ^66^ accounts for anharmonicity. Corresponding vibrational frequencies are found in Table S3 in the SI. Raw electronic energies are listed in Table S1 in the SI.

#### Electronic State Analysis with EOM-CC

Different variants of equation-of-motion coupled cluster methods (EOM-CC)^67^ were employed to provide qualitative insight into the relative energetics between low-lying electronic states. fc-EOMIP-CCSD (ionization potential), starting from the ground electronic state of the closed-shell vinylcyclopentadienyl anion as a reference, was used to compute the lowest-lying neutral radical electronic states. The relative energetics of electronic states in the cation manifold were probed with both fc-EOMEE-CCSD (excitation energy) and fc-EOMSF-CCSD (spin flip) methods using an ANO1 basis set.

## Results

### Generation, Detection, and Spectroscopy of C_7_H_7_ Radicals

Figure 1 shows the PI-TOF-MS of the precursor **mVA** (C_9_H_10_O, *m*/*z* 134), measured both while heating (930 K, top red trace) and without heating (300 K, bottom black trace) the pyrolysis reactor. The only ion counts in the room temperature mass spectrum appear at *m*/*z* 134 and 135, corresponding to photoionization of **mVA** and its ^13^C substituted isotopologue. No signs of dissociative photoionization are seen in the mass spectrum collected at 10.5 eV.

**Figure 1:**
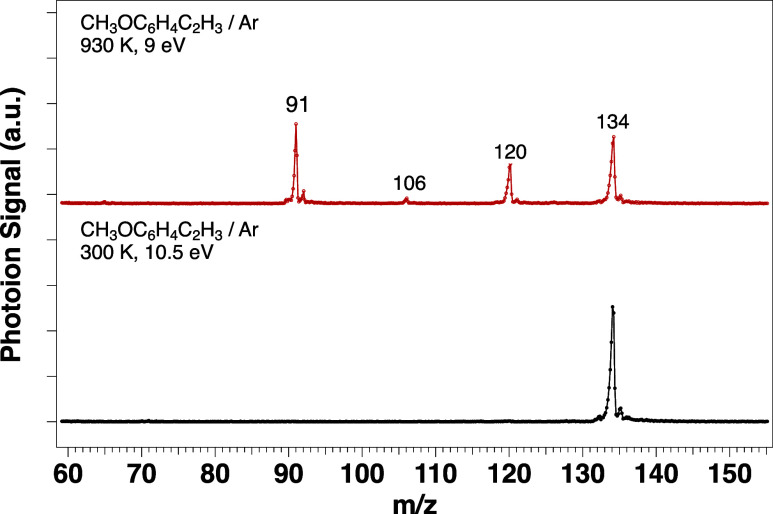
Photoionization time-of-flight mass spectra of *m*-vinylanisole in Ar. The bottom black trace is the mass spectrum at a photon energy of 10.5 eV without heating the microreactor (300 K). The top red trace is the spectrum after heating the microreactor to a temperature of 930 K and using a photon energy of 9 eV.

The red trace in Figure 1 shows the PI-TOF-MS acquired at a photon energy of 9 eV and with the SiC microreactor heated to 930 K. Under these pyrolysis conditions, thermal decomposition product peaks appear at *m*/*z* 120 and 91, while the residual *m*/*z* 134 signal indicates incomplete **mVA** degradation (see Figure S1 for a partial *m*/*z* 134 ms-TPES).^36,68^ Analogous to the known pyrolysis of anisole,^69,70^ the mass spectrum is consistent with initial methyl loss (yielding the C_8_H_7_O *meta-*vinylphenoxy radical, 119 u) followed by CO loss to C_7_H_7_ (91 u) (see Scheme 1). The *m*/*z* 120 peak originates from H addition to 119 u; a partial ms-TPES (Figure S3) of *m*/*z* 120 features an ionization onset in agreement with the calculated AIE of *m-*vinylphenol. The intensity of the target peak at *m*/*z* 91 was optimal at a reactor temperature of 930 K—high enough to maximize conversion of **mVA** and vinylphenoxy but not so high to induce decomposition of C_7_H_7_. Additional discussion of the pyrolysis conditions is found in the Supporting Information.

**Scheme 1:**
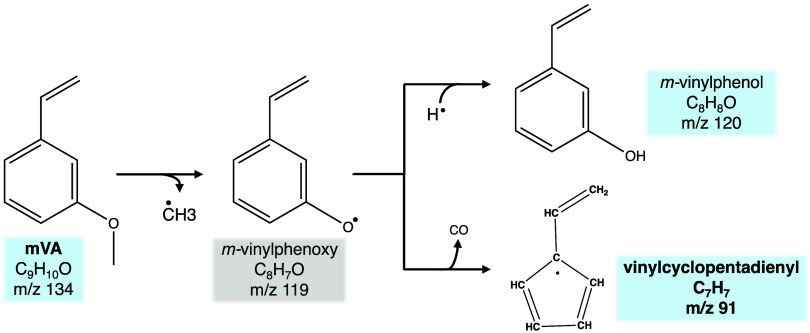
Pyrolysis of *m*-Vinylanisole Proceeds via Methyl Radical Loss, Followed Either by Hydrogen Atom Addition or Sequential Loss of Carbon Monoxide to Produce Vinylcyclopentadienyl

The ms-TPES was constructed by plotting the coincident signal between near-zero kinetic energy electrons and ions of *m*/*z* 91. Previous characterization on this experimental setup demonstrated that inefficient rovibrational cooling in the gas expansion manifests as thermally broadened photoelectron spectra for molecules in the molecular beam.^38^ Ion imaging revealed here that spectral fine structure arises only for photoions born from colder (300 K) rethermalized gas in the detection chamber. The ms-TPES of *m*/*z* 91 shown in Figure 2 is thus extracted solely based on the thermalized ion signal. The full ms-TPES results, including a breakdown of contributions from the molecular beam and background gas ions to the total signal, are provided in Figure S4 of the Supporting Information.

**Figure 2:**
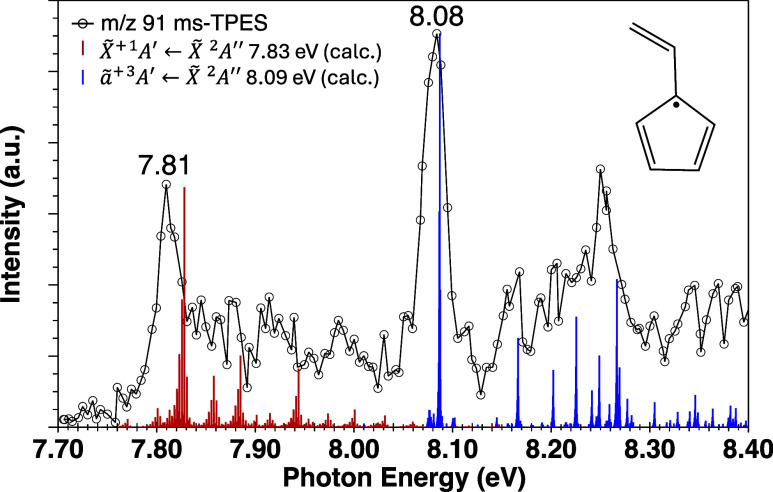
ms-TPES of *m*/*z* 91 based on the 300 K thermalized signal from the pyrolytic flow of CH_3_OC_6_H_4_C_2_H_3_ in Ar at 930 K. Experimental transition energies are noted above origin peaks. Overlaid F–C simulations at 300 K are anchored to the calculated transition energies to the ground electronic state, X̃^+ 1^A′ (red sticks), and first electronic excited state, ã^+ 3^A′ (blue sticks), of vinylcyclopentadienyl cation, respectively.

Three main spectral features are identifiable in the experimental spectrum shown in Figure 2 (circled data points connected by black trace). In order of increasing energy, the first peak lies at (7.81 ± 0.02) eV, with an asymmetric shape consisting of a sharp rise followed by a sloped decay, indicative of an ionization onset followed by an unresolved vibronic progression. A second, sharper and more symmetric peak is at (8.08 ± 0.02) eV. Accounting for the photon energy resolution, Stark shift, the electron kinetic energy resolution, and the rotational envelope at 300 K, we assign ionizing transition energies an uncertainty of 20 meV. Following this second peak is a rise in signal featuring a bump near (8.17 ± 0.02) eV before reaching a third maximum at (8.26 ± 0.02) eV.

### Theory Assigns the ms-TPES of Vinylcyclopentadienyl

Table 1 reports adiabatic ionization energies (AIE) to the two lowest electronic states of the vinylcyclopentadienylium cation, computed using the composite electronic structure scheme described in detail in the Computational Methods. This protocol is conceptually similar to established high-accuracy thermochemical methods such as HEAT^51,52,71–73^ and Wn,^74–78^ in that it decomposes the total relativistic electronic energy into distinct, individually evaluated contributions and benefits from partial error cancellation between terms. However, rather than adhering to a fixed recipe, the present scheme prioritizes computing each energetic contribution with the largest affordable basis set and level of theory.

**Table 1:** Composite Electronic Energy Contributions (in eV) to the Cationic Singlet–Triplet Gap and Adiabatic Ionization Energies

		X̃^+ 1^A′ ← X̃ ^2^A″	ã^+ 3^A′ ← X̃ ^2^A″	ã^+ 3^A′ ← X̃^+ 1^A′
contribution	calculation	adia. IE (X̃^+^)	adia. IE (ã^+^)	ΔS–T
*E*_HF‑SCF_	SCF/pCV6Z	6.6710	6.6330	–0.0379
Δ*E*_fc‑(T)_Λ__^∞^	fc-CCSD(T)_Λ_/pV{Q,5}Z	1.1179	1.4032	0.2853
Δ*E*_fc‑T‑(T)_Λ__	fc-CCSDT – CCSD(T)_Λ_/ANO1	–0.0110	–0.0037	0.0080
Δ*E*_fc‑(Q)_Λ__	fc-CCSDT(Q)_Λ_/ANO0	–0.0017	0.0001	0.0018
Δ*E*_CV_^∞^	(ae – fc)-CCSD/pCV{T,Q}Z	0.0202	0.0168	–0.0034
	+ (ae – fc)-CCSD(T)_Λ_/pCVTZ	–0.0001	0.0020	0.0021
Δ*E*_ZPVE_	fc-CCSD(T)/ANO1	0.0352	0.0387	0.0035
	+ fc-CCSD(T)/ANO0 (VPT2)	0.0035	0.0027	–0.0008
Δ*E*_REL_	ae-CCSD(T)/pCVTZ-unc	–0.0027	–0.0022	0.0005
Δ*E*_DBOC_	fc-CCSD/pVTZ	–0.0039	–0.0038	0.0001
total		7.828	8.087	0.259

Electron correlation effects in this scheme are treated primarily using Λ-based coupled cluster methods.^46,53,54,61^ These approaches provide a more rigorous perturbative correction than conventional CCSD­(T),^44–46^ as they employ a biorthogonal formulation^67^ in which both left- and right-hand eigenvectors of the similarity-transformed Hamiltonian are retained. As a result, Λ-based corrections more closely approximate the fully iterative CCSDT method, particularly in cases where CCSD­(T) is known to overestimate the energetic contribution of triple excitations. Recent benchmark studies have demonstrated that inclusion of Λ-based corrections leads to more reliable higher-order correlation contributions (not limited to only triple excitations, but at any excitation order) in computing energetics,^52,59,79^ structures, and vibrational frequencies.^60,61^

Using this scheme, the adiabatic ionization energy to the lowest singlet electronic state of the cation, X̃^+ 1^A′, is computed to be 7.83 eV, with a conservative estimated uncertainty of ± 0.01 eV. Additional details concerning how the uncertainty was computed are located in the Supporting Information. The first excited state is a triplet, ã^+ 3^A′, and is predicted to lie (0.26 ± 0.01) eV higher in energy, corresponding to an AIE of (8.09 ± 0.01) eV. The electronic character of the neutral and ionized states is illustrated in Figure 3, which shows truncated, qualitative molecular orbital (MO) diagrams^80^ (part a) and the restricted open-shell Hartree–Fock (ROHF) molecular orbitals of the neutral vinylcyclopentadienyl radical (part b). Ionization to the X̃^+ 1^A′ state involves removal of the single unpaired electron from the HOMO (4*a*″), which is largely localized on the *ipso* carbon. Ionization to the ã^+ 3^A′ state instead corresponds to electron removal from the 3*a*″ orbital.

**Figure 3:**
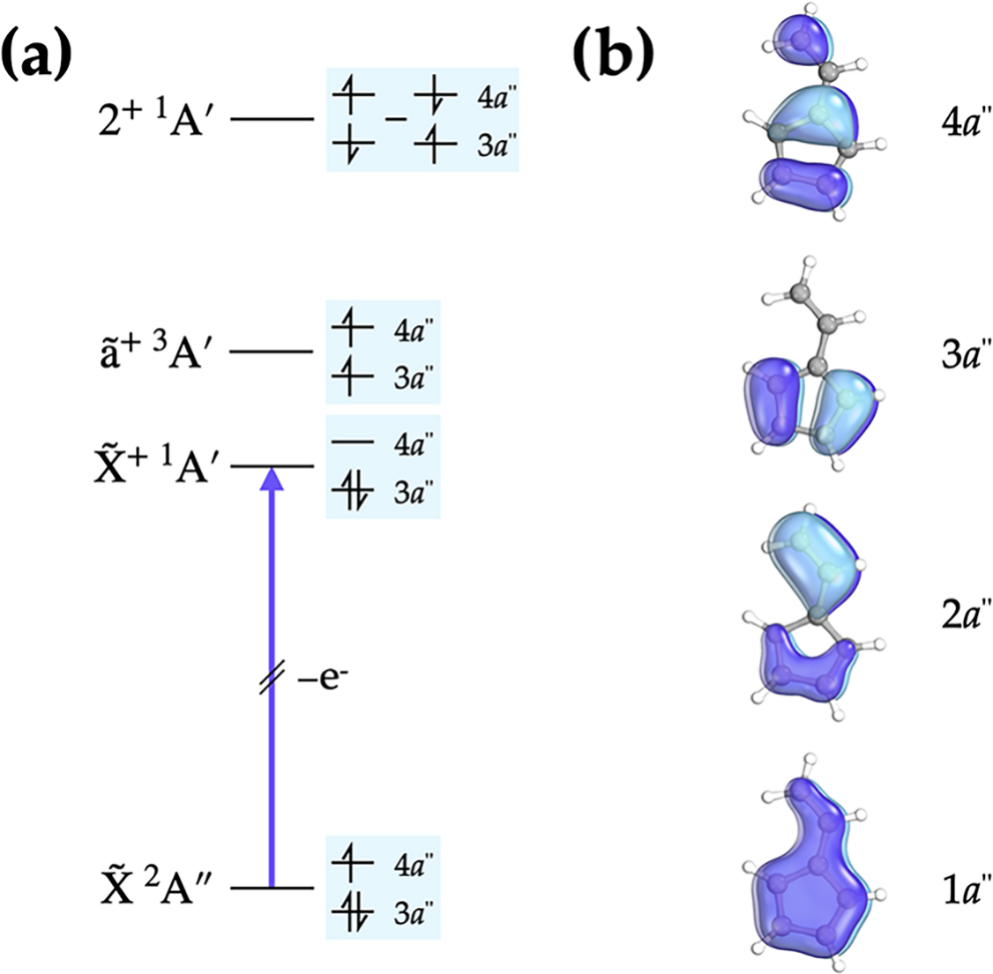
(a) Photoionization of the vinylcyclopentadienyl radical in its ground electronic state to the three lowest cationic electronic states. Light blue insets illustrate truncated molecular orbital diagram descriptions for each state.^80^ (b) Restricted open-shell Hartree–Fock (ROHF) *a*″ molecular orbitals of the ground electronic state of the neutral radical.

Harmonic F–C stick spectra at 300 K are overlaid on the experimental mass-selected threshold photoelectron spectrum in Figure 2, and comparisons to the 0 K simulations are included in the Supporting Information. The 0–0 transition energies of both simulations are fixed at the computed AIEs reported in Table 1; no empirical shifting was applied to improve agreement with experiment. For visual comparison, each simulation was scaled so that the calculated 0–0 transition intensity matched the corresponding experimental origin peak maximum.

The calculated origin of the triplet ã^+ 3^A′ state is in excellent agreement with the experimentally observed peak at 8.08 eV. The computed singlet origin lies slightly higher in energy than the experimental peak identified at 7.81 eV. For comparison, previous CBS-QB3 computations reported in the work of Savee et al.^26^ predict the triplet AIE to be 8.13 eV, approximately 46 meV higher in energy than the second experimental peak and 43 meV higher in energy than the value calculated here, at the upper edge of the CBS-QB3 estimated uncertainty of ≈1 kcal/mol (43 meV).^27^ Their CBS-QB3 singlet AIE lies near 7.94 eV, nearly midway between the first two experimental features and about 130 meV higher than the first experimental peak. Restricted open-shell calculations at ROCBS-QB3,^81^ reported both by Johansson et al.^8^ and Jin et al.,^9^ return a singlet origin of 7.90 eV. The composite singlet AIE computed here, by contrast, is only 18 meV higher in energy than the first peak. In addition to the two lowest-energy peaks, the experimental spectrum exhibits a clear peak near 8.26 eV. In the absence of theoretical guidance, this peak could reasonably be suspected to originate from ionization into a higher-lying electronic state. The F–C simulation in Figure 2, together with computed excited-state energetics summarized in Table 2, indicates that this feature arises from a vibrational progression associated with the ã^+ 3^A′ state rather than from a distinct higher-lying electronic state.

**Table 2:** Lowest Lying Cationic Electronic States, Computed with EOMEE (Excitation Energy) and EOMSF (Spin Flip) at the fc-EOM-CCSD/ANO1 Level

	*E*_EOMEE_ (eV)	*E*_EOMSF_ (eV)	⟨*S* ^2^⟩_EOMSF_
X̃^+ 1^A′			0.02
ã^+ 3^A′	0.395	0.407	2.02
2^+ 1^A′	1.081	0.861	0.02

Optimized equilibrium structures for the neutral radical ground state (X̃ ^2^A″) and the two lowest cationic states, computed at the frozen core CCSD­(T)/ANO1 level, are shown in Figure 4 (Cartesian coordinates are provided in Table S5 of the SI). Structural differences between the neutral and cationic species are modest, but nonetheless the C–C bonds lengthen and contract consistent with the MO diagrams depicting distinct ionization processes from each of the 4*a*″ and 3*a*″ MOs in Figure 3.

**Figure 4:**
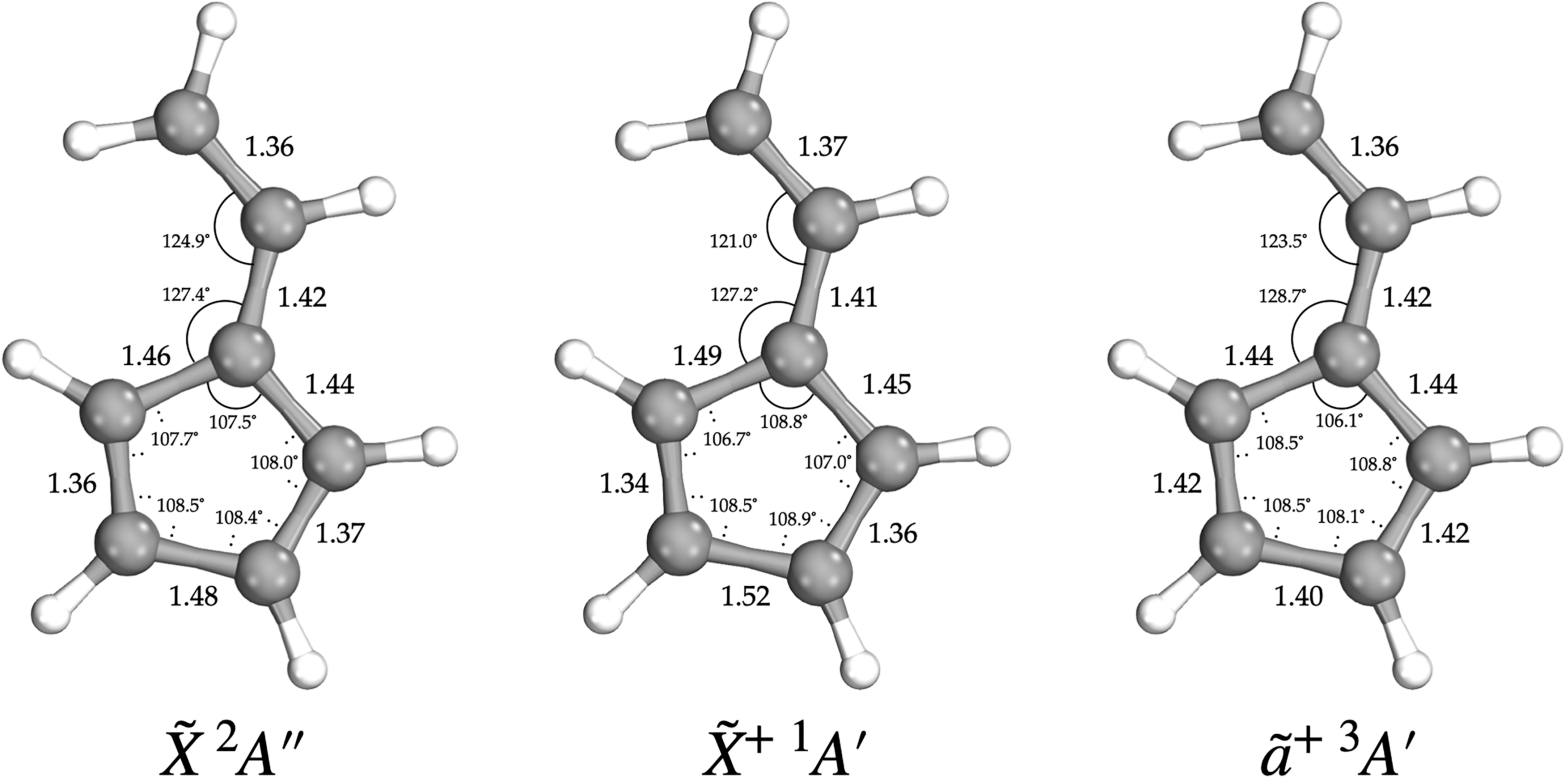
Equilibrium geometries (evaluated at fc-CCSD­(T)/ANO1) for the ground electronic state of the neutral radical (X̃ ^2^A″) and two lowest cation (X̃^+ 1^A′, ã^+ 3^A′) electronic states. C–C bond lengths are reported in Angstroms (Å), bond angles in degrees.

All transitions discussed in this work originate from the ground electronic state (X̃ ^2^A″) of the neutral vinylcyclopentadienyl radical. Equation-of-motion ionization potential (EOMIP) calculations starting from the anion ground state indicate that the first excited state of the neutral radical (2 ^2^A″, here we utilize numeric labeling for states not yet experimentally observed) lies approximately 1.2 eV higher in energy than the ground state at the frozen core EOMIP-CCSD/ANO1 level. Whereas the ground state is characterized by a singly occupied 4*a*″ orbital, the first excited state corresponds to a configuration in which the 3*a*″ orbital is singly occupied and the 4*a*″ is doubly occupied (see Figure 3).

Within the cationic electronic manifold, the three lowest-energy states correspond to the singlet X̃^+ 1^A′, the triplet ã^+ 3^A′, and an open-shell singlet 2^+ 1^A′, as illustrated in Figure 3. To probe their relative energetics, results from both fc-EOMEE-CCSD (excitation energy) and fc-EOMSF-CCSD (spin flip) calculations using an ANO1 basis set are reported in Table 2. Using X̃^+ 1^A′ as the reference state, fc-EOMEE-CCSD places the 2^+ 1^A′ state 1.08 eV higher in energy. On the other hand, fc-EOMSF-CCSD computations, using ã^+ 3^A′ as the reference, estimate this state to lie a more modest 0.86 eV above X̃^+ 1^A′. It is interesting to note, however, that scanning up to 8.85 eV in photon energy (see Figure S5 in the Supporting Information) does not immediately reveal any clear transitions in this region. It is possible that the vertical excitation energies calculated with these two methods do not correspond to electronic states with bound minima; more careful investigation of these electronically excited states would be insightful, but is beyond the scope of the current work. The equation-of-motion spin flip (EOMSF) calculations described here were made possible by the recent implementation of the method for open-shell molecules in the CFOUR program package, with further details to be outlined in a forthcoming publication.

The excellent agreement between the composite prediction for the AIE from X̃ ^2^A″ to the triplet ã^+ 3^A′ state and the experimentally observed peak at 8.08 eV suggests that the influence of the 2 ^2^A″ neutral excited state on the computed triplet AIE is minimal. By contrast, the X̃^+ 1^A′ cationic state is coupled not only to the 2^+ 1^A′ state in Figure 3 and Table 2, but also to an additional, higher-lying singlet state (not shown in Figure 3) characterized by a doubly occupied 4*a*″ orbital and a vacant 3*a*″ orbital (likely 3^+ 1^A′). The challenge of characterizing same-symmetry coupling arises in this three-state singlet manifold, and is expected to have a more pronounced impact on the determination of the singlet AIE and S–T energy gap, consistent with the slightly poorer agreement between theory and experiment for the singlet origin. Supporting this interpretation, inspection of the eigenvector associated with the EOMSF description of the X̃^+ 1^A′ state reveals substantial mixing with the 2^+ 1^A′ configuration. The next higher-lying triplet state, 2^+ 3^A′, is computed at the fc-EOMEE-CCSD/ANO1 level to lie approximately 2.3 eV above the ground electronic state of the cation and is not coupled to any of the lower-lying states discussed here.

The combination of the composite electronic structure calculations and the experimental ms-TPES data enable unambiguous assignment of the *m*/*z* 91 ms-TPE spectrum to the C_7_H_7_ isomer vinylcyclopentadienyl radical within the photon energy range explored in this work. The principal experimental peaks are conclusively attributed to ionization into the X̃^+ 1^A′ cation state at (7.81 ± 0.02) eV with the ã^+ 3^A′ state lying at (8.08 ± 0.02) eV.

## Discussion and Conclusion

With the ms-TPES of vinylcyclopentadienyl in hand, the impact of the vinyl substituent on the electronic structure can be understood by comparing the current results to those of the “bare” cyclopentadienyl radical and its cation. Recalling the earlier description of the ground and first excited states of the neutral vinylcyclopentadienyl radical, it is important to note that these two states form a pseudo Jahn–Teller (JT) coupled pair. They represent the nondegenerate analog to the JT coupled states in the nonsubstituted, centrosymmetric cyclopentadienyl radical. In the case of vinylcyclopentadienyl of *C*_*s*_ symmetry, both states belong to the A″ irreducible representation. The same-symmetry nature of these electronic states unfortunately precludes treatment with our current theoretical methods. As a result, nonadiabatic effects (beyond the inclusion of diagonal Born–Oppenheimer corrections) were not explicitly considered in the present work. In contrast to the JT effect in neutral cyclopentadienyl, the lower symmetry induced by vinyl substitution is expected to weaken the analogous coupling in vinylcyclopentadienyl.

The key influence of the vinyl group is to split the degenerate e_1_^″^ orbitals of the neutral cyclopentadienyl radical to the nondegenerate 3*a*″ and 4*a*″ orbitals in vinylcyclopentadienyl (Figure 3). While the 3*a*″ orbital is lightly stabilized by asymmetric overlap between the ring and the vinyl group, the 4*a*″ orbital has an additional node owing to an antibonding interaction between the ring and vinyl group, raising its energetic ordering to that of the HOMO. Most interesting is that the MO scheme of vinylcyclopentadienyl manifests as a reversal of the energetic ordering of the first singlet and triplet states in the cation manifold relative to the cyclopentadienyl cation (C_5_H_5_^+^: *X̃* ^+ 3^ *A*_2_^′^ < *ã* ^+ 1^ *^1^E*_2_^′^, ΔS–T = 1534 ± 6 cm^–1^ = 0.1902 ± 0.0007 eV),^82^ with the vinylcyclopentadienyl singlet cation being the ground electronic state while the S–T gap is of similar magnitude (C_7_H_7_^+^: *X̃* ^+ 1^ *A*′ < *ã* ^+ 3^ *A*′, ΔS–T = 0.27 ± 0.02 eV). Effectively, while the spin state ordering in cyclopentadienylium can be predicted by considering the exchange energy benefiting stabilization of the triplet versus the JT-lowered singlet, the substantial MO splitting inherent to vinylcyclopentadienyl lends itself to a more straightforward prediction of the S–T ordering and MO scheme.

The close proximity of the ground singlet and triplet states of the vinylcyclopentadienyl cation is in stark contrast to C_7_H_7_ isomers tropyl and benzyl, offering an interesting physical insight relevant to resolving isomeric detection and quantification in gas phase mixtures. The schematic in Figure 5 shows the ionizing transitions of the four detected C_7_H_7_ isomers to date. The energetic ordering of the cation ground electronic states (all singlets) for the series is tropyl, benzyl, vinylcyclopentadienyl (from lowest to highest energy). This is understood from the now complete measurement of the ionization energies across the series and previously compiled calculations for the relative energetics of the doublet neutrals: with benzyl referenced at 0 kJ/mol, tropyl and vinylcyclopentadienyl lie 69 and 89 kJ/mol higher, respectively.^12^ Detection of the neutral species for this series of seven-, six-, and five-membered ring species can be contextualized by the S–T gaps of the cations. The tropyl cation’s low IE of 6.23 eV owes to the drastic lowering of the singlet cation state as a D_7h_ Hückel aromatic (relative to the antiaromatic triplet state and JT distorted doublet neutral).^25^ Consequently, the S–T gap for tropyl is the highest, near 3 eV.^25^ A near 2 eV splitting is present in benzyl, such that while both of those C_7_H_7_ species have significantly lower IEs than vinylcyclopentadienyl (6.23 eV for tropyl and 7.25 eV for benzyl), the nearest triplet states are both above 9 eV.^24,25^ The C_7_H_7_ isomer, 3-ethynylcyclopentenyl (**3ecp**) radical, is an RSR that has been spectroscopically characterized within electrical discharges, but has not yet been experimentally implicated within combustion-relevant C_7_H_7_ mixtures.^83^ Despite being a high-energy isomer (CBS-QB3 calculations put it 167 kJ/mol above benzyl), **3ecp** has been proposed for consideration in reactions of H atom additions to fulvenallenyl or vinylpropargyl + acetylene. The ionization energy of **3ecp** was determined at 6.93 eV and is predicted to have a similarly high S–T gap of 2+ eV, the latter value based on DFT calculations reported by Reilly et al.^83^ (note we keep their label *T*_1_^+^ for the first triplet state in Figure 5). This leaves a detection window in between the three S–T gaps ca. 8 eV spectrally clear for characterization of vinylcyclopentadienyl using transitions to *both* its singlet and triplet cation states. Such spectroscopic access to multiple electronic states even at medium resolution is expected to be helpful in studies seeking to differentiate and even quantify the presence of vinylcyclopentadienyl radical within a mixture of C_7_H_7_ isomers.

**Figure 5:**
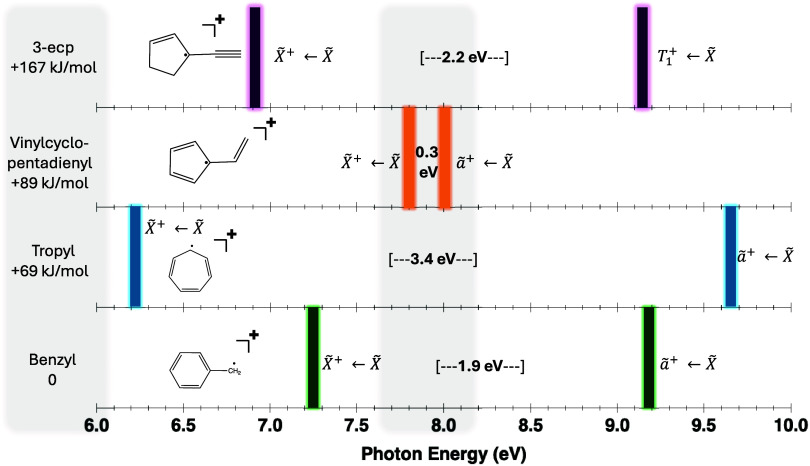
Schematic of the valence ionization of the four detected C_7_H_7_ isomers to date. The ionizing transitions are denoted by colored vertical lines, indicating where ion signal is expected in photoionization based experiments. All IE values are experimental as reported in this work (vinylcyclopentadienyl) or referenced in the main text (benzyl, tropyl, and **3ecp**), except the S–T gap of 3-ethynylcyclopentenyl, which was calculated using DFT. The relative energetics of the neutrals are also compiled based on previous calculations referenced in the text.

We have generated the C_7_H_7_ aromatic, resonance-stabilized vinylcyclopentadienyl radical via the pyrolysis of a C_9_H_10_O precursor, *m*-vinylanisole, in a SiC microreactor. The reaction mixture was probed using *i* ^2^PEPICO spectroscopy. The ms-TPES of the *m*/*z* 91 radical of C_7_H_7_ composition reveals ionization into both the lowest singlet and triplet states of the cation, with ionization energies of (7.81 ± 0.02) eV and (8.08 ± 0.02) eV, respectively. The singlet–triplet gap in the vinylcyclopentadienyl cation is thus determined by experiment to be 0.27 eV. Independent high-level *ab initio* composite calculations yield adiabatic ionization energies of (7.83 ± 0.01) eV and (8.09 ± 0.01) eV, respectively, providing reliable assignment of the experimental origins. These spectroscopic signatures can be used to identify this radical in reaction mixtures with isomer-specificity. This work demonstrates the advantages of combining sophisticated experimental and theoretical approaches when interpreting spectra of resonance-stabilized radicals with a multitude of isomers and close-lying electronic states.

## Supplementary Material





## Notes

The SI contains a discussion of the pyrolysis of meta-vinylanisole (mVA) and report of the ms-TPE spectra for additional species observed in the mass spectra at m/z 120 and 134; The x/y data points of all the processed experimental data reported both in the main text and supporting information are available in the accompanying spreadsheet (XLSX)
Additional information on the ms-TPES of *m*/*z* 91, including ion imaging results and a second ms-TPES over a longer energy range, is compiled. Subsequently, we include a PI spectrum of vinylcyclopentadienyl and a comparison to previously reported PI spectra.^9,11^ Next, we present evidence of C_7_H_7_ isomerization from vinylcyclopentadienyl to benzyl radical in the following section. Additional computational results are tabulated: computed raw electronic energies, harmonic frequencies, zero-point vibrational corrections, diagonal Born–Oppenheimer corrections, and equilibrium structures (PDF)

## #

Deceased March 21, 2025.
