# Supplementary material for: The Threshold Photoelectron Spectrum of the Vinylcyclopentadienyl Radical, a C7H7 Resonance-Stabilized Radical
Source: J Phys Chem A. 2026 Jun 9;130(24):4583–93. doi: 10.1021/acs.jpca.6c02174 (PMC13288686; doi:10.1021/acs.jpca.6c02174)
Supplement: Supplementary file 2 [file jp6c02174_si_002.pdf]

Supporting Information to: The Threshold Photoelectron Spectrum of the Vinylcyclopentadienyl Radical, a C_7H_7 Resonance-Stabilized Radical

Rory McClish,[†,‡] Megan R. Bentley,[¶] Mia Muse,[†,‡] Gregory H. Jones,[¶] Peter R. Franke,[¶] Domenik Schleier,[§] Patrick Hemberger,[‖] Andras Bodi,[‖] G. Barney Ellison,[‡] John F. Stanton,[¶,⊥] and Jordy Bouwman[*,†,‡]

†*Laboratory for Atmospheric and Space Physics, University of Colorado, Boulder, Colorado 80303, United States*

‡*Department of Chemistry, University of Colorado, Boulder, Colorado 80309, United States*

¶*Quantum Theory Project, Department of Chemistry, University of Florida, Gainesville, Florida 32611, United States*

§*Institut für Physik und Astronomie, Technische Universität Berlin, Hardenbergstrasse 36, Berlin 10623, Germany.*

‖*Laboratory for Synchrotron Radiation and Femtochemistry, Paul Scherrer Institute, 5232 Villigen, Switzerland*

⊥*Deceased March 21, 2025*

E-mail: jordy.bouwman@colorado.edu

Additional Experimental Results

Pyrolysis of *m*-Vinylanisole

In scanning the photon energy to measure the ms-TPES of m/z 91 (vinylcyclopentadienyl) in the heated flow of **mVA**, we also obtain the partial ms-TPES of ion peaks at m/z 134 and 120 (also from considering only the rethermalized signal). In these cases, we have calculated the AIE for candidate species using CBS-QB3 for the closed-shell singlets and ROCBS-QB3 for the open-shell doublets using the Gaussian 16.[1] Figure S1 shows the ms-TPES of m/z 134 between 7.7–8.4 eV. Despite the present photon energy range and resolution, signal onset occurs above 7.95 eV and the band origin appears to be near 8.06 eV. While a reference ms-TPES is not experimentally known for vinylanisole, we note that the present ms-TPE spectrum is qualitatively similar to the initial ms-TPES structure of analogous species anisole (methoxybenzene, $C_6H_4{-}O{-}CH_3$) and styrene (vinylbenzene).[2] The anisole family is susceptible to complex photoelectron spectra due to an extended FC envelope from geometry differences between the neutral and cation.[3] Considering the expected accuracy of (RO)CBS-QB3 is on the order of ≈0.05 eV (note the greater discrepancy with experiment, ≈0.09 eV using RO and ≈0.13 eV for unrestricted CBS-QB3, for vinylcyclopentadienyl radical as discussed in the main text), the calculated AIE of 3-vinylanisole agrees with the spectrum of the precursor.[4,5]

The use of **mVA** as a pyrolytic precursor to vinyl substituted cyclopentadienyl radical is inspired by the known decomposition chemistry of anisole.[6,7] Anisole undergoes stepwise loss of methyl and carbon monoxide to produce the cyclopentadienyl radical:

$$C_6H_4{-}O{-}CH_3 \longrightarrow C_6H_4{-}O^{\bullet} + CH_3^{\bullet}$$

$$C_6H_4{-}O^{\bullet} \longrightarrow C_5H_5^{\bullet} + CO$$

The pulsed-flow microreactor setups used by the Ellison group helped to preserve the phenoxy radical,[6,7] while greater conversion to the phenol has been observed in continuous flow experiments.[8] However, in the latter case, careful optimization of the pyrolysis conditions have allowed for measurement of the phenoxy radical ms-TPES.[8] All three studies observe a small amount of secondary bimolecular chemistry from methyl addition to the cyclopentadienyl radical:

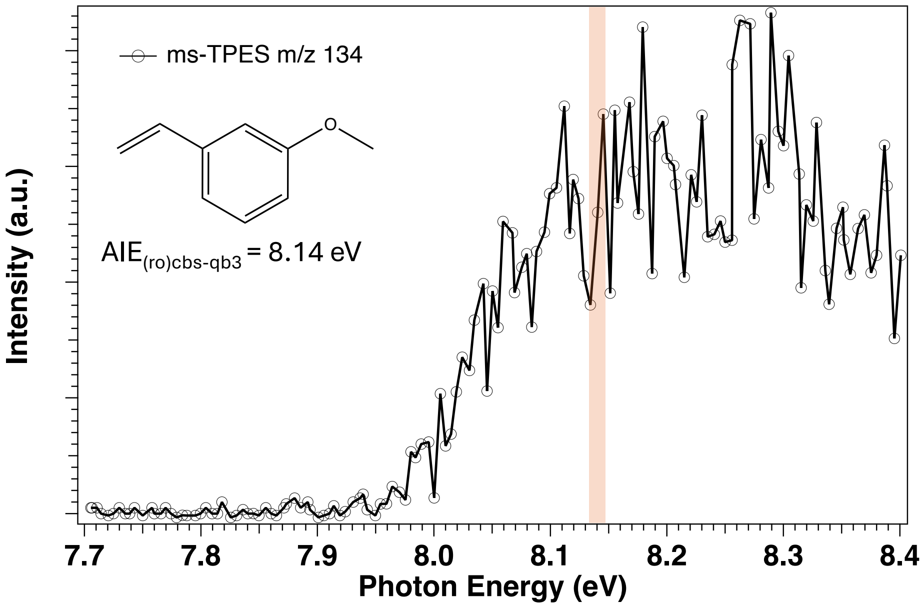

Figure S1: The ms-TPES of the thermalized m/z 134 signal, with calculated (RO)CBS-QB3 AIE values for **mVA** denoted on the spectrum with an orange shaded bar. The ms-TPES is constructed from the same scan of **mVA**/Ar taken at 930 K used to measure vinylcyclopentadienyl.

$$C_5H_5^{\cdot} + CH_3^{\cdot} \longrightarrow C_5H_5{-}CH_3$$

Figure 1 in the main text (reproduced here as Fig. S2) indicates that **mVA** pyrolysis follows the same unimolecular decomposition steps. Methyl loss from **mVA** at 134 u nominally yields the vinylphenoxy radical ($C_2H_3{-}C_6H_4{-}O^{\cdot}$, 119 u):

$$C_2H_3{-}C_6H_4{-}O{-}CH_3 \longrightarrow C_2H_3{-}C_6H_4{-}O^{\cdot} + CH_3^{\cdot}$$

However, we do not observe any m/z 119, suggesting that despite the short residence time of the gas in the SiC microreactor, the vinylphenoxy radical is not preserved under our experimental conditions. Instead, we conclude that it either decomposes to vinylcyclopentadienyl (signal at m/z 91) or undergoes H-addition to vinylphenol ($C_2H_3{-}C_6H_4{-}OH$, 120 u):

$$C_2H_3{-}C_6H_4{-}O^{\cdot} \longrightarrow C_2H_3{-}C_5H_4^{\cdot} + CO$$

$$C_2H_3{-}C_6H_4{-}O^{\cdot} + H^{\cdot} \longrightarrow C_2H_3{-}C_6H_4{-}OH$$

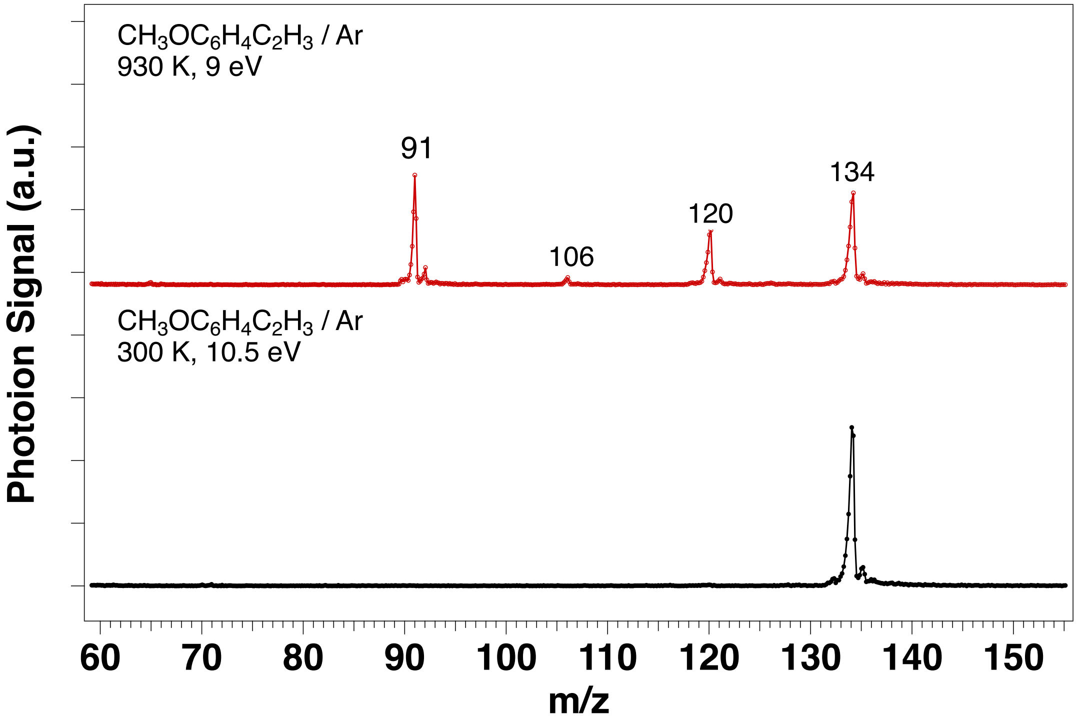

Figure S2: Figure 1 of the main text. Photoionization time-of-flight mass spectra of m-vinylanisole in Ar. The bottom black trace is the mass spectrum at a photon energy of 10.5 eV without heating the microreactor (300 K). The top red trace is the spectrum after heating the microreactor to a temperature of 930 K and using a photon energy of 9 eV.

Figure S3 demonstrates that the m/z 120 signal we observe can be attributed to m-vinylphenol considering that the calculated AIE of 8.33 eV is just past the signal onset. The ms-TPES suggests an experimental IE of about 8.3 eV. Beyond similar considerations in the assignment of **mVA**, we do not rule out further isomerization of C_8H_8O, but note this is not the focus of this work.

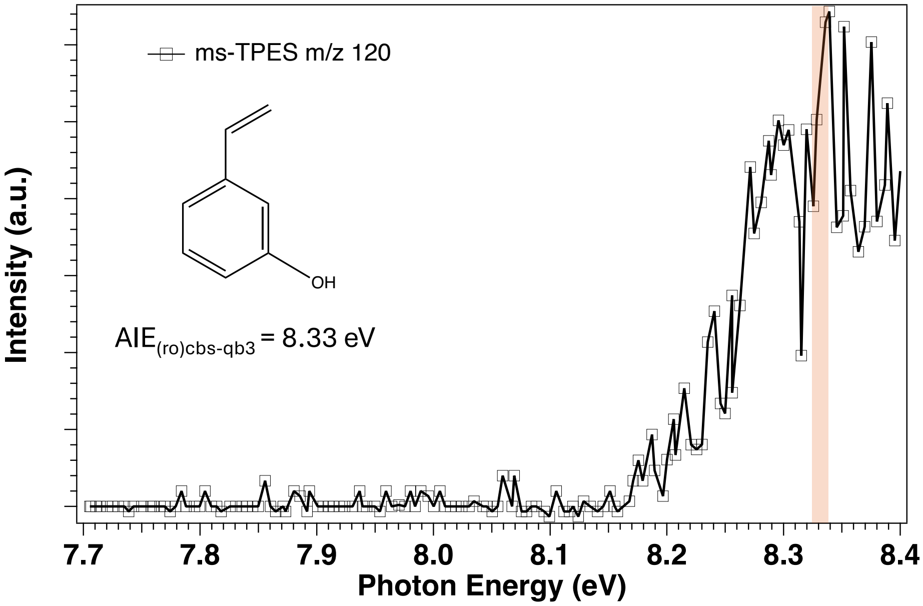

Figure S3: The ms-TPES of m/z 120 based on the rethermalized signal, with the calculated (RO)CBS-QB3 AIE for *meta*-vinylphenol denoted on the spectrum with an orange shaded bar. The ms-TPES is constructed from the same scan of **mVA**/Ar taken at 930 K used to measure vinylcyclopentadienyl.

Additional Results Pertaining to the Ion Signal at m/z 91

Ion-imaging of ms-TPES with i^2PEPICO is discussed in the Methods. Figure S4 shows the m/z 91 ms-TPES constructed with and without selecting for signal originating from the molecular beam or the rethermalized gas. Qualitatively, the blue total-ion trace is the sum of the black (background gas) and red (molecular beam) traces. Consistent with previous characterization of this setup,[9] species in the molecular beam are rovibrationally hot (roughly the temperature of the gas in the microreactor) and thus the spectral features are washed out via thermal broadening. The spectrum is recovered by selecting the ion counts associated with the rethermalized (300 K) background gas (black trace, open circles, the same as in Figure 2 of the main text).

We also conducted a longer scan of the photon energy using a slightly lower flow rate of 10 sccm

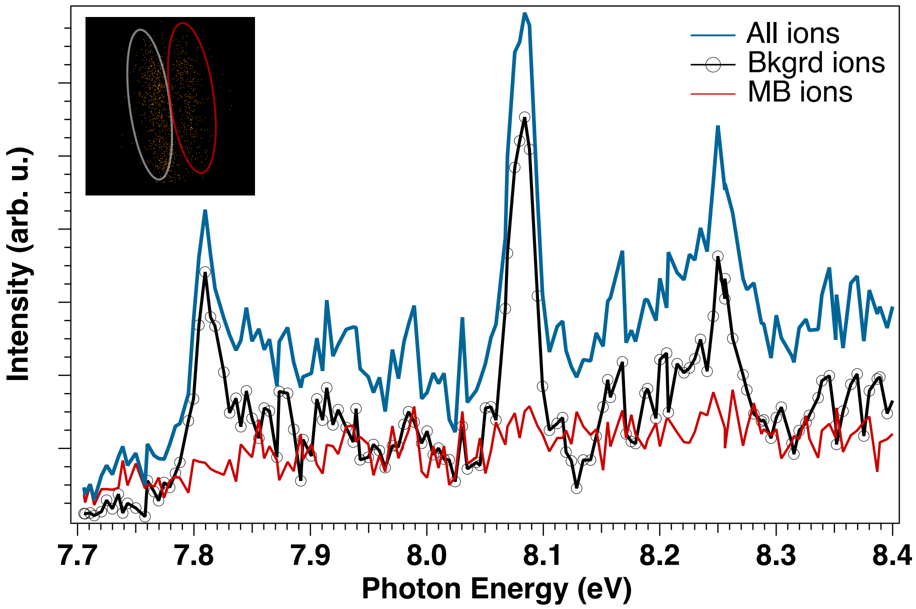

Figure S4: Ion-imaging study of the m/z 91 ms-TPES. The blue trace is the ms-TPES constructed with all ions and between 7.7–8.4 eV with a stepsize of 5 meV and an integration time per step in photon energy of 120 s. The black trace with open circles at the individual data points is the ms-TPES reported in the main text and is sampled only from the background gas ions, shown qualitatively in the light grey oval in the inset of a representative ion detector image of m/z 91 counts. The red ms-TPES trace is from selecting the counts associated with the molecular beam (within the red oval region of the inset image). The cation velocity map has been defocused here to obtain higher mass resolution in the TOF mass spectrum.

(versus 14 sccm in the 7.7–8.4 eV scan) and a shorter signal integration time of 60 s (versus 120 s). The scan was on the same heated flow of **mVA** in Ar at 930 K. The background ms-TPES of m/z 91 from both scans is shown in Fig. S5. Per the discussion in the main text, since the longer scan range extends from 7.5–8.85 eV, this scan offers some insight into the energy difference between $2^+\,{}^1A'$ and $\tilde{X}^+\,{}^1A'$. In the case of the fc-EOMSF-CCSD calculation, there is no clear ionizing transition in the experimental ms-TPES near 8.7 eV. The scan does not go quite far enough to decisively comment on the fc-EOMEE-CCSD calculation placing the $2^+\,{}^1A'$ near 8.9 eV.

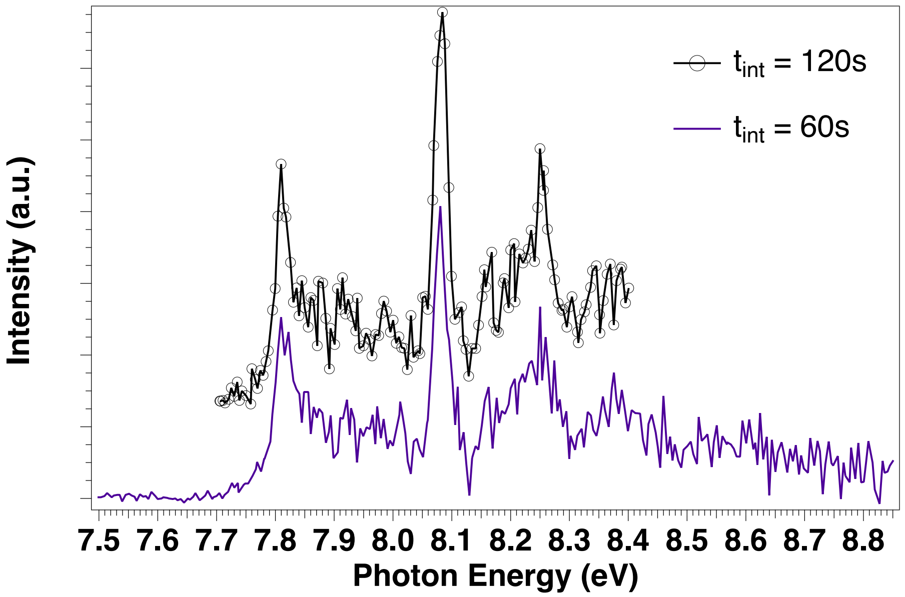

Figure S5: The reproducibility of the m/z 91 ms-TPES when only considering the ions stemming from the rethermalized pyrolysate. The bottom purple trace is the background ms-TPES over 7.5–8.85 eV with a stepsize of 5 meV and an integration time per step in photon energy of 60 s. The flow rate of **mVA**/Ar was 10 sccm. The top black trace with open circles at each point is the background ms-TPES from 7.7–8.4 eV with a stepsize of 5 meV and an integration time per step in photon energy of 120 s. The flow rate of **mVA**/Ar for recording the black trace was increased to 14 sccm.

We extract total ion yield spectra of vinylcyclopentadienyl from our data to enable a comparison to the PI spectrum measured by Jin et al.[10] and Shahanand et al.[11]. The present spectra are shown in Fig S6 and are constructed by relaxing the threshold energy criterion for photoelectrons by considering the coincidences of photoelectrons with kinetic energies up to ≈0.7 eV. Our resulting PI spectra are thus valid up to about 8.4 eV (+0.7 eV above the origin), and we consider all three ion-imaging cases (all ions, background gas only, molecular beam only) as included in Fig. S6. Additionally, we overlay the data points of Jin et al.[10] as reported in their supporting information

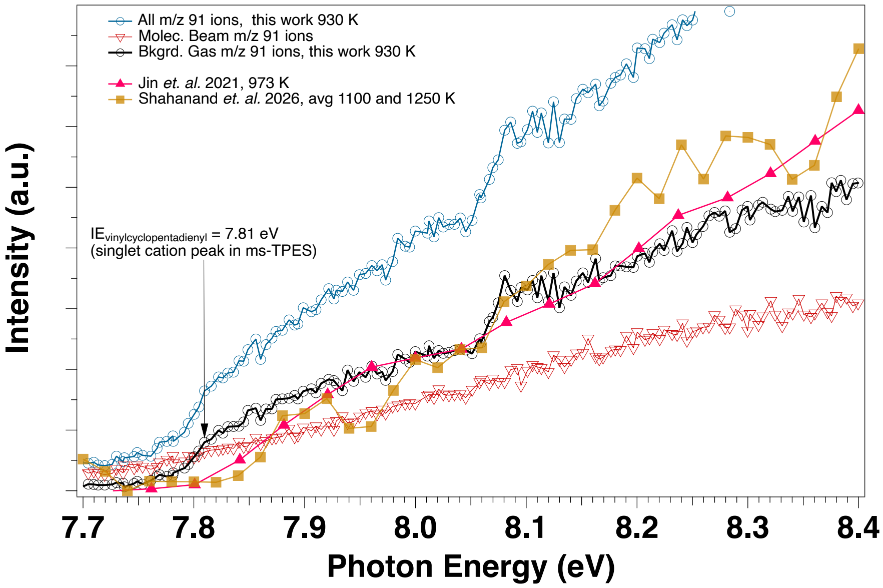

Figure S6: The PI spectrum of m/z 91 constructed considering photoelectrons up to $\sim$0.7 eV under three ion-imaging scenarios. Blue open circles is the spectrum using all m/z 91 ions, the black trace with open circles is from only the background gas, and the red triangles is only from counts within the molecular beam. The data points from Jin et al.[10] are shown as connected pink solid triangles. The averaged PI curve of Shahanand et al.[11] is in solid gold squares. Both literature spectra are arbitrarily scaled to match the room temperature PI spectrum of this work.

(scaled arbitrarily and purely for visual comparison with our all-ion PI spectrum). We also plot the average photoionization cross section as measured by Shahanand et al.[11]. We posit that the physical underpinnings of the differences between the spectra are twofold. First, both of the literature spectra are PI curves that have been normalized to the measured photon flux from the respective synchrotrons as a function of photon energy, whereas the photon flux was not measured concurrently here. Changes in the photon flux across photon energies will modulate the intensity of ion counts in a nonlinear fashion, thereby affecting the slope of the PI spectrum. However, this effect across the relatively short photon energy range here is likely to be minor. Second, ion-imaging enabled by i^{2}PEPICO has revealed that the gas flow in the reactor determines the subsequent expansion and thus the thermal distribution of neutral species in the sampled molecular beam. Moreover, considering the partition of total ion counts between the (hot) molecular beam and the rethermalized (300 K) gas in the detection chamber dictates that sampling effects are particularly important to consider when measuring vinylcyclopentadienyl. Neither of the PI spectra collected at the HLS II and the ALS specifically screen for the source of ions, that is, they are composite spectra of counts

arising from both the molecular beam and background gas, from which the extent of rovibrational cooling in the molecular beam is uncertain. Between the two main differences, normalization to photon flux and sampling effects, we suspect that within this photon energy range, sampling effects will likely dominate the differences observed between the PI spectrum reported here and those in the literature, but emphasize the overall qualitative agreement between the two. This implies that the use of vinylanisoles to be broadly applicable under a range of pyrolysis and flow conditions. Overall, using these PI curves should enable sufficient fitting of experimental m/z 91 PI spectra from reaction studies and help discern the presence of vinylcyclopentadienyl in reactive mixtures.

Rearrangement of Vinylcyclopentadienyl to Benzyl radical

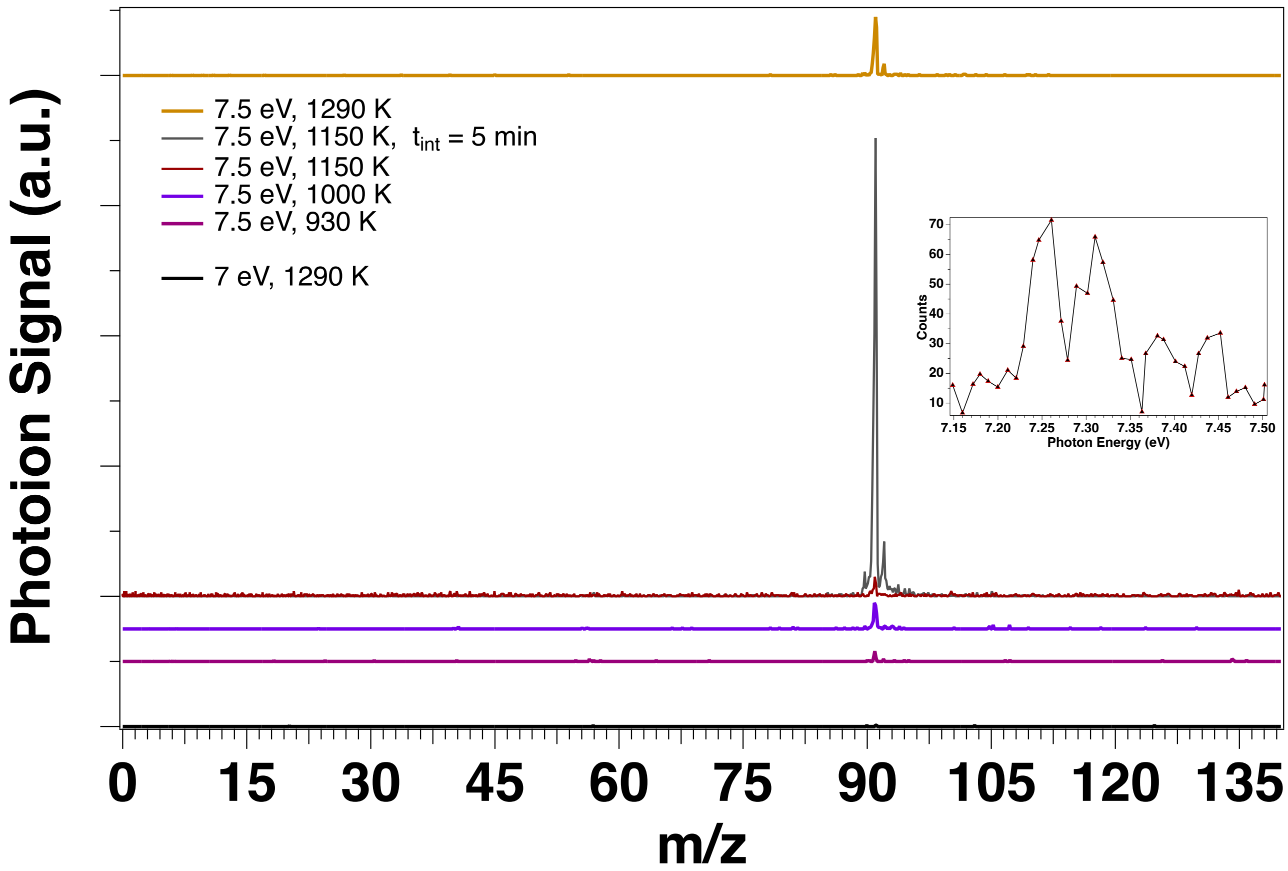

Figure S7: Photoionization mass spectra of m–vinylanisole measured at multiple temperatures, photon energies, and integration times. Inset ms-TPES of m/z 91 confirms the minor production of benzyl radical.

In light of the C_7H_7 PES as calculated by Martí et al.[12] and Meng et al.[13], we checked for

evidence of vinylcyclopentadienyl isomerization as low as 930 K. Figure S7 summarizes the results. The mass spectra were recorded at different photon energies and pyrolysis temperatures, with a 7 eV, 1290 K spectrum on the bottom showing no sign of a m/z 91 species (ruling out the C_7H_7 isomers tropyl and **3ecp** which have IEs of 6.23 and 6.93 eV, respectively).[14,15] At 7.5 eV and 930 K, a few counts of m/z 91 are collected (a peak height of only 8 counts), the intensity of which slightly increases at 1000 and 1290 K.

To boost the low photon energy m/z 91 counts, we increased the integration time from one (red 1290 K trace) to five minutes (gray 1290 K trace). We conducted a short scan of the photon energy to measure an ms-TPES at 1290 K over the well-known benzyl resonances, and—as seen in the inset of the figure—we are able to resolve two peaks in agreement with the first two transitions within the ground state ionization $\tilde{X}^+ \leftarrow \tilde{X}$ of benzyl (see Savee et al.[16]). To our knowledge, this is the first experimental evidence supporting the reaction path identified between vinylcyclopentadienyl and benzyl identified by Martí et al.[12] and Meng et al.[13].

Supporting Computational Data

Franck-Condon Simulations at Different Temperatures

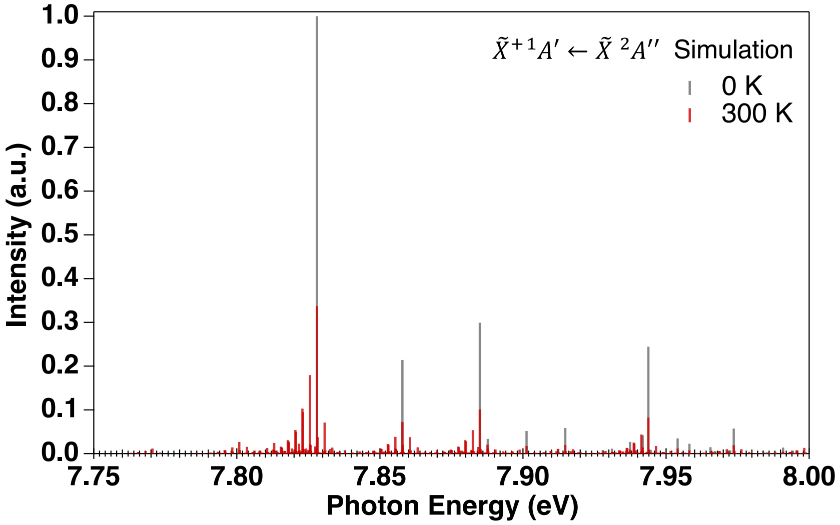

Figure S8: Franck-Condon simulation to the singlet state of the vinylcyclopentadienyl cation at 0 K and 300 K.

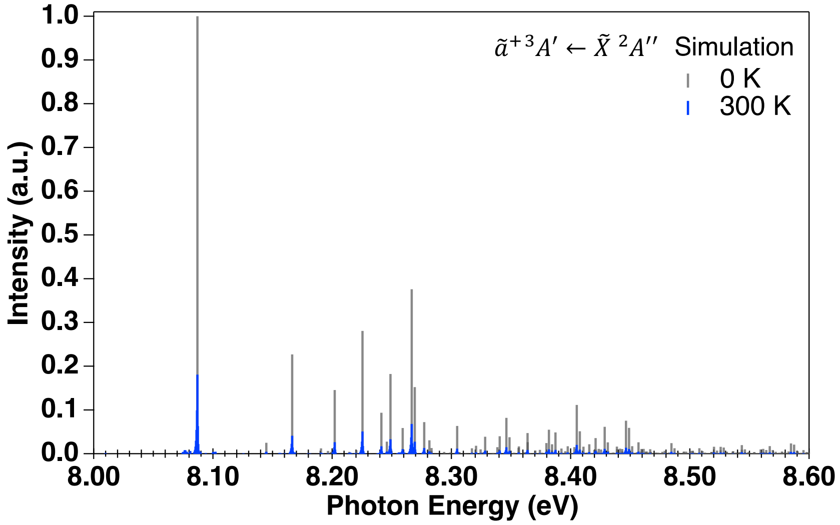

Figure S9: Franck-Condon simulation to the triplet state of the vinylcyclopentadienyl cation at 0 K and 300 K.

Composite calculations by component

Table S1: Raw electronic energies (in a.u.) performed at the equilibrium structure specified. Nonrelativistic post-SCF contributions are reported as correlation energies.

Calculation	$\tilde{X}\,^2A''$	$\tilde{X}^+\,^1A'$	$\tilde{a}^+\,^3A'$
Structure	fc-(T)/ANO1 [ROHF]	fc-(T)/ANO1 [RHF]	fc-(T)/ANO1 [ROHF]
SCF/pVDZ	-269.091151	-268.847273	-268.849877
SCF/pVTZ	-269.157953	-268.913863	-268.915372
SCF/pVQZ	-269.174677	-268.929933	-268.931336
SCF/pV5Z	-269.178699	-268.933587	-268.934985
SCF/pCVDZ	-269.094173	-268.849995	-268.852433
SCF/pCVTZ	-269.159448	-268.915342	-268.916784
SCF/pCVQZ	-269.175472	-268.930718	-268.932117
SCF/pCV5Z	-269.178878	-268.933769	-268.935165
SCF/pCV6Z	-269.179293	-268.934139	-268.935533
SCF/ANO0	-269.147056	-268.900627	-268.903058
SCF/ANO1	-269.171110	-268.925636	-268.927166
fc-CCSD/pVDZ	-0.965817	-0.934367	-0.926210
fc-CCSD/pVTZ	-1.141799	-1.104348	-1.096337
fc-CCSD/pVQZ	-1.196888	-1.157818	-1.149659
fc-CCSD/pV5Z	-1.214354	-1.174796	-1.166550
fc-CCSD/pCVDZ	-0.973908	-0.942509	-0.934333
fc-CCSD/pCVTZ	-1.148042	-1.110540	-1.102520
fc-CCSD/pCVQZ	-1.199596	-1.160511	-1.152345
ae-CCSD/pCVDZ	-1.234169	-1.202562	-1.194445
ae-CCSD/pCVTZ	-1.493826	-1.455812	-1.447884
ae-CCSD/pCVQZ	-1.577240	-1.537509	-1.529455
fc-CCSD(T)/pVDZ	-1.007273	-0.976748	-0.966766
fc-CCSD(T)/pVTZ	-1.201865	-1.164057	-1.153938
fc-CCSD(T)/pVQZ	-1.261742	-1.221898	-1.211605
fc-CCSD(T)/pV5Z	-1.280829	-1.240349	-1.229957
fc-CCSD(T)$_\Lambda$/pVDZ	-1.005400	-0.974848	-0.964869

Continued on next page

Table S1 continued from previous page

Calculation<br>Structure	$\tilde{X}\,^2A''$<br>fc-(T)/ANO1 [ROHF]	$\tilde{X}^+\,^1A'$<br>fc-(T)/ANO1 [RHF]	$\tilde{a}^+\,^3A'$<br>fc-(T)/ANO1 [ROHF]
fc-CCSD(T)$_\Lambda$/pVTZ	-1.199160	-1.161375	-1.151274
fc-CCSD(T)$_\Lambda$/pVQZ	-1.258838	-1.219039	-1.208762
fc-CCSD(T)$_\Lambda$/pV5Z	-1.277868	-1.237442	-1.227064
fc-CCSD(T)$_\Lambda$/ANO0	-1.039041	-1.007089	-0.997075
fc-CCSD(T)$_\Lambda$/ANO1	-1.214126	-1.175602	-1.165471
fc-CCSDT/ANO0	-1.042010	-1.010396	-1.000039
fc-CCSDT/ANO1	-1.216635	-1.178514	-1.168074
fc-CCSDT(Q)$_\Lambda$/ANO0	-1.045520	-1.013969	-1.003545
fc-CCSD(T)$_\Lambda$/pCVDZ	-1.014435	-0.983923	-0.973903
fc-CCSD(T)$_\Lambda$/pCVTZ	-1.206052	-1.168193	-1.158074
ae-CCSD(T)$_\Lambda$/pCVDZ	-1.275871	-1.245155	-1.235156
ae-CCSD(T)$_\Lambda$/pCVTZ	-1.554362	-1.515994	-1.505891
ae-CCSD(T)/pCVTZ-unc	-270.735384	-270.452670	-270.444012
ae-CCSD(T)/pCVTZ-unc//SFX2C1e	-270.839181	-270.556566	-270.547889

End of Table S1

Uncertainty estimates of composite calculations

Broadly speaking, the total uncertainty on the computed adiabatic ionization energies and singlet-triplet gap is taken to be the Euclidean norm of assumed-independent uncertainties associated with each term in the composite recipe. In particular, uncertainties reported here are expressed in terms of basis set incompleteness of the HF-SCF energy and the various correlation energy increments. The uncertainty in the HF-SCF energy is determined by the difference between the HF-SCF energy evaluated with the largest basis set affordable (here, cc-pCV6Z) and that determined by the next largest basis set in the same family (cc-pCV5Z). For correlation energies, if only cc-pVDZ (or ANO0) was tractable, for example, the uncertainty is the correlation contribution itself. If cc-pVTZ (ANO1) is tractable, the uncertainty then is the difference between the correlation increment evaluated with cc-pVTZ and cc-pVDZ. (or ANO1-ANO0) For extrapolated contributions, the uncertainty is the difference between the extrapolated value (e.g. cc-pV{T,Q}Z) and the raw value evaluated with only the larger basis set (in this example, cc-pVQZ, also called the "extrapolation distance"). If a basis set beyond cc-pVQZ is affordable, the uncertainty is the difference between the largest extrapolated value (cc-pVX-1, XZ) and the next largest extrapolated value (cc-pVX-2, X-1Z). As shown in Table SS2, this procedure is followed for all correlation contributions and the core-valence contribution.

Regarding scalar relativity, the X2C1e model is quite rigorous, and nothing higher level (relativistically speaking) was accessible to us for the species studied here. The choice of ROHF precluded us from applying spin-free Dirac-Coulomb (SFDC) theory. Similarly, CCSD(T) electron correlation is sufficiently high level for the evaluation of a relativistic correction. We assumed that those choices did not contribute to the overall uncertainty. Uncertainty due to basis set incompleteness was quantified as the difference between CCSD(T)/cc-pCVTZ-unc and CCSD(T)/cc-pCVDZ-unc relativistic corrections. Determining uncertainties on the remaining auxiliary corrections takes a slightly different approach.

Due to nearby low-lying excited electronic states, there is some uncertainty with regard to the coupling between nuclear motion and the electronic wavefunction. To quantify the rough order of magnitude of this effect, we calculated the DBOC at the CCSD/cc-pVTZ level. We take the entire value as an estimate of the uncertainty for this term.

The dominant sources of uncertainty in the ZPVE are the level of theory of the harmonic ZPVE and the VPT2 description of the anharmonic effects. A smaller source of uncertainty is the level of theory used for the VPT2 correction. We quantify the uncertainty in the harmonic ZPVE as the difference between the CCSD(T)/ANO1 and CCSD(T)/ANO0 values. For the uncertainty in VPT2, we take the VPT2 correction itself. We ignore the uncertainty associated with the level of theory of the anharmonic corrections.

When the Euclidean norm is taken of the uncertainties on the individual contributions to the composite ionization energies, we arrive at a value of ≈ 66 cm^{-1} (or 8 meV) for the ionization energy between the ground state doublet radical and the ground state singlet cation. The uncertainty computed for the ionization energy to the triplet state of the cation is smaller, at 53 cm^{-1}, and the singlet-triplet gap between the two cationic states smaller still, at 26 cm^{-1}. To account for any possible underestimations of uncertainty in terms where such quantification is less

straightforward, we choose to round the largest computed theoretical uncertainty to 10 meV (or $\approx$ 80 cm^{-1}), and use this value to represent a conservative uncertainty estimate for all ionization energies and the singlet-triplet gap computed in this work.

Table S2: (in cm^{-1}).

Contribution	Calculation (Increment)	$\tilde{X}^{+\,1}A' \leftarrow \tilde{X}\,^2A''$	$\tilde{a}^{+\,3}A' \leftarrow \tilde{X}\,^2A''$	$\tilde{a}^{+\,3}A' \leftarrow \tilde{X}^{+\,1}A'$
$E_{\text{HF-SCF}}$	SCF/pCV6Z − SCF/pCV5Z	10	10	<1
$\Delta E^{\infty}_{\text{fc-(T)}_\Lambda}$	fc-$(T)_\Lambda$/pV{Q,5}Z − fc-$(T)_\Lambda$/pV{T,Q}Z	40	23	17
$\Delta E_{\text{fc-T - (T)}_\Lambda}$	fc-(T) - $(T)_\Lambda$/ANO1 − fc-(T) - $(T)_\Lambda$/ANO0	14	9	3
$\Delta E_{\text{fc-(Q)}_\Lambda}$	fc-$(Q)_\Lambda$/ANO0	14	1	15
$\Delta E^{\infty}_{\text{CV}}$	(ae − fc)-CCSD/pCV{T,Q}Z			
	− (ae − fc)-CCSD/pCVQZ	21	18	3
ΔE_{ZPVE}	fc-CCSD(T)/ANO1	8	18	10
	+ fc-CCSD(T)/ANO0 (VPT2)	28	22	6
ΔE_{REL}	ae-CCSD(T)/pCVTZ-unc	2	2	<1
ΔE_{DBOC}	fc-CCSD/pVTZ	32	31	1
	SQRT(SUMSQ) (cm^{-1})	66	53	26

End of Table S2

Frequency computations

Table S3: fc-CCSD(T)/ANO1 harmonic frequencies (ω) and ZPVE in cm^{-1}. The anharmonic correction to the harmonic ZPVE, obtained by a VPT2 computation using a quartic force field of fc-CCSD(T)/ANO0 quality, is included at the bottom of the table.

Mode	Sym	$\tilde{X}\,^2A''$	$\tilde{X}^+\,^1A'$	$\tilde{a}^+\,^3A'$
		ω	ω	ω
1	A'	3254	3275	3266
2	A'	3250	3265	3261
3	A'	3242	3264	3249
4	A'	3227	3248	3240
5	A'	3219	3237	3235
6	A'	3165	3204	3192
7	A'	3156	3163	3163
8	A'	1574	641	1584
9	A'	1545	584	1534
10	A'	1484	1569	1470
11	A'	1444	1496	1449
12	A'	1409	1395	1412
13	A'	1309	1323	1388
14	A'	1291	1314	1306
15	A'	1278	1303	1303
16	A'	1173	1165	1244
17	A'	1093	1121	1117
18	A'	1062	1109	1075
19	A'	1021	1016	1056
20	A'	987	934	1027
21	A'	904	882	928
22	A'	728	679	836
23	A'	622	591	641
24	A'	464	459	466
25	A'	219	241	222
26	A"	997	1089	1001

Continued on next page

Table S3 continued from previous page

Mode	Sym	$\tilde{X}\,^2A''$	$\tilde{X}^+\,^1A'$	$\tilde{a}^+\,^3A'$
		ω	ω	ω
27	A”	903	1006	985
28	A”	891	970	959
29	A”	888	941	909
30	A”	765	844	835
31	A”	713	750	770
32	A”	674	671	625
33	A”	538	487	452
34	A”	523	403	441
35	A”	229	187	228
36	A”	123	103	120
ZPVE		24682	24966	24994
VPT2//fc-CCSD(T)/ANO0		-325	-297	-304

End of Table S3

Table S4: Calculated diagonal Born-Oppenheimer (DBOC) corrections to the $\tilde{X}\,^2A''$ electronic state of the vinylcyclopentadienyl radical (in a.u.and eV), evaluated at the fc-(T)/ANO1 [ROHF] equilibrum geometry.

DBOC	[a.u.]	[meV]
SCF/pVDZ	0.0135	0.3670
SCF/pVTZ	0.0138	0.3759
SCF/pVQZ	0.0139	0.3781
fc-MP2/pVDZ	0.0136	0.3691
fc-MP2/pVTZ	0.0140	0.3803
fc-CCSD/pVDZ	0.0135	0.3662
fc-CCSD/pVTZ	0.0143	0.3899

End of Table S4

Equilibrium Geometries

Table S5: Equilibrium geometries optimized with fc-CCSD(T)/ANO1 (in bohr).

Molecule	Atom	X	Y	Z
C_7H_7 $\tilde{X}\,^2A''$	C	-0.51621612	0.42927893	0.00000000
	C	0.61083334	-2.09386907	0.00000000
	C	3.16882334	-1.80765187	0.00000000
	C	3.75415345	0.92111320	0.00000000
	C	1.52257768	2.23588823	0.00000000
	C	-3.13309122	1.05118529	-0.00000000
	C	-5.05195432	-0.66329589	-0.00000000
	H	-0.42032329	-3.85324948	-0.00000000
	H	4.56139695	-3.30048349	0.00000000
	H	5.63610062	1.71325676	0.00000000
	H	1.28456644	4.26260172	0.00000000
	H	-3.60001083	3.04962354	-0.00000000
	H	-7.00555828	-0.06034125	-0.00000000
	H	-4.68459781	-2.67642490	-0.00000000
$C_7H_7^+$ $\tilde{X}^+\,^1A'$	C	-0.52011294	0.45538803	0.00000000
	C	0.56323827	-2.13675301	0.00000000
	C	3.09141658	-1.87381464	0.00000000
	C	3.71736813	0.93485889	0.00000000
	C	1.53303598	2.27367851	0.00000000
	C	-3.09930488	1.12162184	-0.00000000
	C	-4.94074602	-0.69058171	-0.00000000
	H	-0.50104898	-3.87253315	-0.00000000
	H	4.49077129	-3.35927192	0.00000000
	H	5.61691503	1.68745101	0.00000000
	H	1.29463718	4.29799383	0.00000000
	H	-3.59842370	3.10725196	-0.00000000

Continued on next page

Table S5 continued from previous page

Molecule	Atom	X	Y	Z
	H	-6.92443829	-0.17937403	-0.00000000
	H	-4.48501948	-2.68642914	-0.00000000
$C_7H_7^+$ $\tilde{a}^+$ $^3A'$	C	-0.56297290	0.42130698	0.00000000
	C	0.58853574	-2.04030242	0.00000000
	C	3.26324578	-1.73283149	0.00000000
	C	3.81134843	0.86384185	0.00000000
	C	1.48173470	2.20986155	0.00000000
	C	-3.16944708	1.05455491	-0.00000000
	C	-5.06185148	-0.69930787	-0.00000000
	H	-0.39156496	-3.83168948	-0.00000000
	H	4.62637567	-3.25229166	0.00000000
	H	5.67088311	1.70488928	0.00000000
	H	1.26736851	4.24233278	0.00000000
	H	-3.64475174	3.04762190	-0.00000000
	H	-7.02237174	-0.11834139	-0.00000000
	H	-4.68039179	-2.71081787	-0.00000000

End of Table S5

References

(1) Frisch, M. J.; Trucks, G. W.; Schlegel, H. B.; Scuseria, G. E.; Robb, M. A.; Cheeseman, J. R.; Scalmani, G.; Barone, V.; Petersson, G. A.; Nakatsuji, H. et al. Gaussian 16 Revision C.02. 2016; Gaussian Inc. Wallingford CT.

(2) Hemberger, P.; Pan, Z.; Wu, X.; Zhang, Z.; Kanayama, K.; Bodi, A. Photoion Mass-Selected Threshold Photoelectron Spectroscopy to Detect Reactive Intermediates in Catalysis: From Instrumentation and Examples to Peculiarities and a Database. *J. Phys. Chem. C* **2023**, *127*, 16751–16763.

(3) Eisenhardt, C. G.; Gemechu, A. S.; Baumgärtel, H.; Chelli, R.; Cardini, G.; Califano, S. Excited state photoelectron spectroscopy of anisole. *Phys. Chem. Chem. Phys.* **2001**, *3*, 5358–5368.

(4) Simmie, J. M.; Somers, K. P. Benchmarking compound methods (CBS-QB3, CBS-APNO, G3, G4, W1BD)

against the Active Thermochemical Tables: A litmus test for cost-effective molecular formation enthalpies. *J. Phys. Chem. A* **2015**, *119*, 7235–7246.

(5) Wood, G. P. F.; Radom, L.; Petersson, G. A.; Barnes, E. C.; Frisch, M. J.; Montgomery, J., John A. A restricted-open-shell complete-basis-set model chemistry. *J. Chem. Phys.* **2006**, *125*, 094106.

(6) Friderichsen, A. V.; Shin, E.-J.; Evans, R. J.; Nimlos, M. R.; Dayton, D. C.; Ellison, G. B. The pyrolysis of anisole (C6H5OCH3) using a hyperthermal nozzle. *Fuel* **2001**, *80*, 1747–1755.

(7) Scheer, A. M.; Mukarakate, C.; Robichaud, D. J.; Ellison, G. B.; Nimlos, M. R. Radical Chemistry in the Thermal Decomposition of Anisole and Deuterated Anisoles: An Investigation of Aromatic Growth. *J. Phys. Chem. A* **2010**, *114*, 9043–9056.

(8) Fernholz, C.; Bodi, A.; Hemberger, P. Threshold Photoelectron Spectrum of the Phenoxy Radical. *J. Phys. Chem. A* **2022**, *126*, 9022–9030.

(9) Hemberger, P.; Wu, X.; Pan, Z.; Bodi, A. Continuous Pyrolysis Microreactors: Hot Sources with Little Cooling? New Insights Utilizing Cation Velocity Map Imaging and Threshold Photoelectron Spectroscopy. *J. Phys. Chem. A* **2022**, *126*, 2196–2210, PMID: 35316066.

(10) Jin, H.; Xing, L.; Liu, D.; Hao, J.; Yang, J.; Farooq, A. First aromatic ring formation by the radical-chain reaction of vinylacetylene and propargyl. *Combust. Flame* **2021**, *225*, 524–534.

(11) Shahanand, J.; Bhattacharyya, S.; Lemmens, A. K.; Dias, N.; Ahmed, M.; Wolf, T. J. A.; Bambha, R. P.; Michelsen, H. A. Comparative study of anisole and 4-vinylanisole pyrolysis: Insight into char, tar, and particle formation during lignin pyrolysis. *PRX Energy* **2026**, *5*, 023005.

(12) Martí, C.; Michelsen, H. A.; Najm, H. N.; Zádor, J. Comprehensive Kinetics on the C7H7 Potential Energy Surface under Combustion Conditions. *J. Phys. Chem. A* **2023**, *127*, 1941–1959, PMID: 36802584.

(13) Meng, Q.; Chi, Y.; Zhang, L.; Zhang, P. On non-hydrogen-atom products of thermal decomposition of benzyl radical: A theoretical investigation by the transition state theory/multi-well master equation approach. *Int. J. Chem. Kinet.* **2024**, *56*, 571–583.

(14) Fischer, K. H.; Hemberger, P.; Bodi, A.; Fischer, I. Photoionisation of the tropyl radical. *Beilstein J. Org. Chem.* **2013**, *9*, 681–688.

(15) Reilly, N. J.; Kokkin, D. L.; Ward, M. L.; Flores, J.; Ross, S. D.; McCaslin, L. M.; Stanton, J. F. Gas-Phase Optical Detection of 3-Ethynylcyclopentenyl: A Resonance-Stabilized C7H7 Radical with an Embedded 1-Vinylpropargyl Chromophore. *J. Am. Chem. Soc.* **2020**, *142*, 10400–10411.

(16) Savee, J. D.; Zádor, J.; Hemberger, P.; Sztáray, B.; Bodi, A.; Osborn, D. L. Threshold photoelectron spectrum of the benzyl radical. *Molecular Physics* **2015**, *113*, 2217–2227.
